# Supplementary material for: Structure and Bonding in Amorphous Red Phosphorus
Source: Angew Chem Int Ed Engl. 2023 May 5;62(24):e202216658. doi: 10.1002/anie.202216658 (PMC10952455; doi:10.1002/anie.202216658)
Supplement: Supplementary file 1 — Supporting Information [file ANIE-62-0-s002.pdf]

Angewandte Chemie
Eine Zeitschrift der Gesellschaft Deutscher Chemiker

Supporting Information

Structure and Bonding in Amorphous Red Phosphorus

*Y. Zhou, S. R. Elliott, V. L. Deringer**

Computational Methods

GAP-driven melt-quench simulations. A general-purpose ML potential, introduced in Ref. [S1], was used to generate structural models of a-P. For this potential, the Gaussian Approximation Potential (GAP) framework[S2] was used, together with the Smooth Overlap of Atomic Positions (SOAP)[S3] structural descriptor. The reference database, from which the potential had "learned", contains configurations of various crystalline, nanostrucured, and liquid phases, and random structures generated via a GAP-driven random structure-searching protocol.[S4] The reference data (energies, forces, and stresses) had been computed at the PBE+MBD level.

We performed GAP-driven MD simulations in the NPT ensemble, using LAMMPS,[S5] with a Nosé–Hoover thermostat[S6] controlling the temperature and a barostat[S7] controlling the external pressure. Melt-quench simulations were used to generate small-scale a-P models, starting from three metastable liquid configurations taken from the reference database of the original potential paper.[S1] These models each contain 248 atoms with mass densities of 2.5, 2.5, and 2.4 g cm^{-3}, respectively; their structures represent different stages of the liquid-to-liquid phase transition.[S8] Both P molecules and covalently connected networks were observed in the former two models, and the third almost fully resembles the network liquid without any P tetrahedra. The models were rapidly de-compressed from ≈ 1 to 0 GPa and annealed at 1,500 K over 10 ps, and then quenched to 1,200 K at a rate of 10^{13} K s^{-1}. After that, the structural models were slowly cooled down from 1,200 K to 100 K at a rate of 10^{11} K s^{-1}, thereby forming amorphous networks (Figure S1a). During quenching, the number of fully three-coordinated atoms steadily increased (Figure S1b). We have previously shown that this process allows the simulation to explore the relevant configurational space, leading to a structural model consistent with previous experimental data (including structure factors at ambient and high pressure; see Ref. [S9] and references therein). The a-P models were further relaxed successively using GAP and DFT (PBE+D3). A force tolerance of 0.01 eV Å^{-1} was used as the stopping criterion in GAP-based relaxations.

Structural models of hydrogenated a-P. To obtain structural models of hydrogenated a-P (a-P:H) without coordination defects, over-coordinated (N = 4) P atoms in the pristine models were removed, leading to the formation of additional two-coordinated P atoms. An exception to this exists when an N = 4 atom is bonded to an N = 2 atom. Instead of removing the N = 4 atom, which leads to an N = 1 atom with a dangling P–P bond, the N = 2 atom was removed. After that, one hydrogen atom was added to each of the under-coordinated (N = 2) P atoms, placed perpendicularly to the two chemical bonds of that P atom, on the side with more open space (*i.e.* with the lowest sum of distances to all neighbouring P atoms in the local environment), forming a short P–H bond. The initial bond length was set to 1.4 Å, a typical value found in phosphorus hydrides.[S10] The resultant structures were then computationally optimised using PBE+D3.

DFT computations. Structural relaxations and single-point calculations were performed using the Vienna Ab initio Simulation Package (VASP)[S11] with projector augmented-wave (PAW)[S12] pseudopotentials. The PBE+D3 method[S13] was used for structural relaxations, whereas the HSE06 hybrid functional[S14] with many-body dispersion (MBD) corrections[S15] was used in the subsequent static computations, based on pre-converged PBE wave functions. The plane-wave energy cut-off was 500 eV, the energy tolerance for SCF convergence was 10^{-7} eV per cell, and the force tolerance for structural relaxation was 0.01 eV Å^{-1}. Gaussian smearing with a width of 0.05 eV was used to determine partial occupancies during SCF cycles.

In structural relaxation, all lattice parameters and atomic coordinates were optimised. A *k*-point grid with a largest allowed spacing of 0.1 Å^{-1} along each reciprocal lattice vector was used for structural relaxation for crystalline modifications, and a grid spacing of 0.15 Å^{-1} (*i.e.*, a lower *k*-point density) was used for the following HSE06 computations of the electronic structure and bonding, to reduce computational cost. The resulting *k*-point grids are given in Table S1. We verified that changing between spacings of 0.1 and 0.15 Å^{-1} did not change the predicted band gap of black phosphorus (0.23 eV) within the quoted accuracy. For a-P and a-P:H models, all data reported are from Γ-point-only computations (corresponding to a “1 1 1” grid in Table S1).

Effect of zero-point vibrations. We tested the effect of zero-point vibrational energy (ZPVE) and report the results in Table S4. The ZPVE estimation is based on a finite-difference approach for phonon computations and was carried out using VASP. We found that this procedure was not feasible at the HSE06+MBD level, and so – just like for the structural relaxations – we used the less computationally demanding, yet still accurate PBE+D3 level. The results in Table S4 clearly show that the energy ordering is retained when ZPVE estimates are included: the amorphous-phase models **1** to **3** are more stable than white P, and slightly less so than the nanorod models *n*1 to *n*3. The ranking of the individual a-P models is also consistent with HSE06+MBD (*cf.* Table S2), with **1** being least stable and **3** most stable among the three.

Chemical bonding. The Local-Orbital Basis Suite Towards Electronic-Structure Reconstruction (LOBSTER) code[S16] was used to project the self-consistent wave functions onto an auxiliary basis of localised, atom-centered orbitals (3s and 3p on each P atom), enabling crystal orbital Hamilton population (COHP) analysis.[S17]

Visualisation. The structural models in Figure 1a and 3a in the main text, as well as the fragments shown in Figure 2a, were visualised using OVITO;[S18] the visualisations of local motifs and band-resolved charge density in Figure 3c were created using VESTA.[S19]

Supplementary Tables

Table S1. Lattice parameters of fully DFT-optimised structural models of phosphorus modifications, obtained by structural optimisation at the PBE+D3 level as described in the Computational Methods section above. We also list the *k*-point grids for computations using PBE+D3 (resulting from the use of a grid spacing of 0.1 Å^{-1}) and HSE06+MBD (grid spacing 0.15 Å^{-1}), respectively. Computations for the a-P structural models used the Γ point only ("1 1 1").

	Lattice parameters (optimised using PBE+D3)						*k*-point grids	
	a (Å)	*b* (Å)	*c* (Å)	α (°)	β (°)	γ (°)	PBE+D3	HSE06+MBD
White (β-P_4)	5.548	10.967	11.115	94.570	99.422	100.498	12 6 6	8 4 4
**1**	17.912	17.752	17.822	90.707	90.327	91.113	1 1 1	1 1 1
**2**	17.867	17.710	17.768	91.591	90.944	90.918	1 1 1	1 1 1
**3**	17.600	17.733	17.837	89.224	89.881	91.005	1 1 1	1 1 1
**1H**	18.237	17.804	18.207	90.275	91.459	90.588	1 1 1	1 1 1
**2H**	18.522	17.964	17.910	91.366	88.372	91.745	1 1 1	1 1 1
**3H**	17.736	17.969	18.214	89.996	91.329	91.478	1 1 1	1 1 1
*n*1	10.974	10.177	10.206	90.000	83.014	90.000	6 7 7	4 5 5
*n*2	12.826	14.021	6.816	90.000	118.591	90.000	6 5 11	4 3 7
*n*3	11.600	6.890	16.581	90.000	103.469	90.000	6 10 4	4 7 3
violet	9.239	9.140	22.238	90.000	97.939	90.000	7 7 3	5 5 2
fibrous	12.318	13.009	7.145	116.667	106.286	97.920	6 6 11	4 4 8
black	3.308	10.673	4.418	90.000	90.000	90.000	19 6 15	13 4 10

Table S2. Energetics of the a-P models **1** to **3** and comparison to crystalline phases, computed using HSE06+MBD. Raw data, given in eV per simulation cell, were taken from the VASP output. The energy data were then converted into units of eV atom^{-1} (by dividing by the number of atoms in the cells) and kJ mol^{-1}. The relative energies (ΔE) are referenced to white phosphorus. The rightmost column shows the values that are given in Table 1 of the main text.

	VASP computed energy		ΔE (E_x – E_{white})	
	(eV cell^{-1})	(eV atom^{-1})	(eV atom^{-1})	(kJ mol^{-1})
White (β-P_4)	–155.0493	–6.4604	± 0 (reference)	
**1**	–1624.7432	–6.5514	–0.0910	-8.8
**2**	–1629.1749	–6.5693	–0.1089	–10.5
**3**	–1633.2137	–6.5855	–0.1252	–12.1
*n*1	–317.0399	–6.6050	–0.1446	–14.0
*n*2	–317.9779	–6.6245	–0.1642	–15.8
*n*3	–370.2597	–6.6118	–0.1514	–14.6
violet	–559.5045	–6.6608	–0.2004	–19.3
fibrous	–279.6255	–6.6578	–0.1974	–19.0
black	–53.2733	–6.6592	–0.1988	–19.2

Table S3. Energy contributions from many-body dispersion (MBD) corrections for the different phases of phosphorus considered. This table gives only the MBD part, and the listed contribution refers to the total energy. For example, for white P, the VASP total energy is –6.4604 eV atom^{-1} (Table S2), and the MBD contribution to this energy is –0.1424 eV atom^{-1}. The rightmost column shows the values that are given in Table 1 of the main text.

	VASP computed dispersion (MBD) contribution		
	(eV cell^{-1})	(eV atom^{-1})	(kJ mol^{-1})
White (β-P_4)	–3.4183	–0.1424	–13.7
**1**	–42.0909	–0.1697	–16.4
**2**	–42.4837	–0.1713	–16.5
**3**	–42.4939	–0.1713	–16.5
*n*1	–7.9305	–0.1652	–15.9
*n*2	–8.3810	–0.1746	–16.8
*n*3	–9.5392	–0.1703	–16.4
violet	–14.9247	–0.1777	–17.1
fibrous	–7.4739	–0.1780	–17.2
black	–1.6529	–0.2066	–19.9

Table S4. Energetics of the a-P models **1** to **3** and comparison to crystalline phases, computed using PBE+D3. The total energy per cell has been corrected by adding the zero-point vibrational energy (E_{ZPVE}; computed using a finite-difference approach) to the total electronic energy (E_0; from static SCF calculations). The relative energies (ΔE) are referenced to white phosphorus.

	PBE+D3 energy E_0 (eV cell^{-1})	Zero-point vibrational energy E_{ZPVE} (eV cell^{-1})	Corrected total energy E (E_0 + E_{ZPVE})		ΔE (E_x – E_{white}) including ZPVE	
			(eV cell^{-1})	(eV atom^{-1})	(eV atom^{-1})	(kJ mol^{-1})
White (β-P_4)	–129.3537	1.0969	–128.2569	–5.3440	± 0 (reference)	
**1**	–1353.5873	12.5739	–1341.0134	–5.4073	–0.0633	–6.1
**2**	–1357.5485	12.6610	–1344.8874	–5.4229	–0.0789	–7.6
**3**	–1361.1570	12.7383	–1348.4187	–5.4372	–0.0931	–9.0
*n*1	–263.9897	2.4979	–261.4918	–5.4477	–0.1037	–10.0
*n*2	–264.8879	2.4762	–262.4118	–5.4669	–0.1229	–11.9
*n*3	–308.5154	2.8961	–305.6193	–5.4575	–0.1135	–10.9
violet	–466.3045	4.4613	–461.8431	–5.4981	–0.1541	–14.9
fibrous	–233.0237	2.2168	–230.8069	–5.4954	–0.1514	–14.6
black	–44.3532	0.3927	–43.9605	–5.4951	–0.1510	–14.6

Supplementary Figures

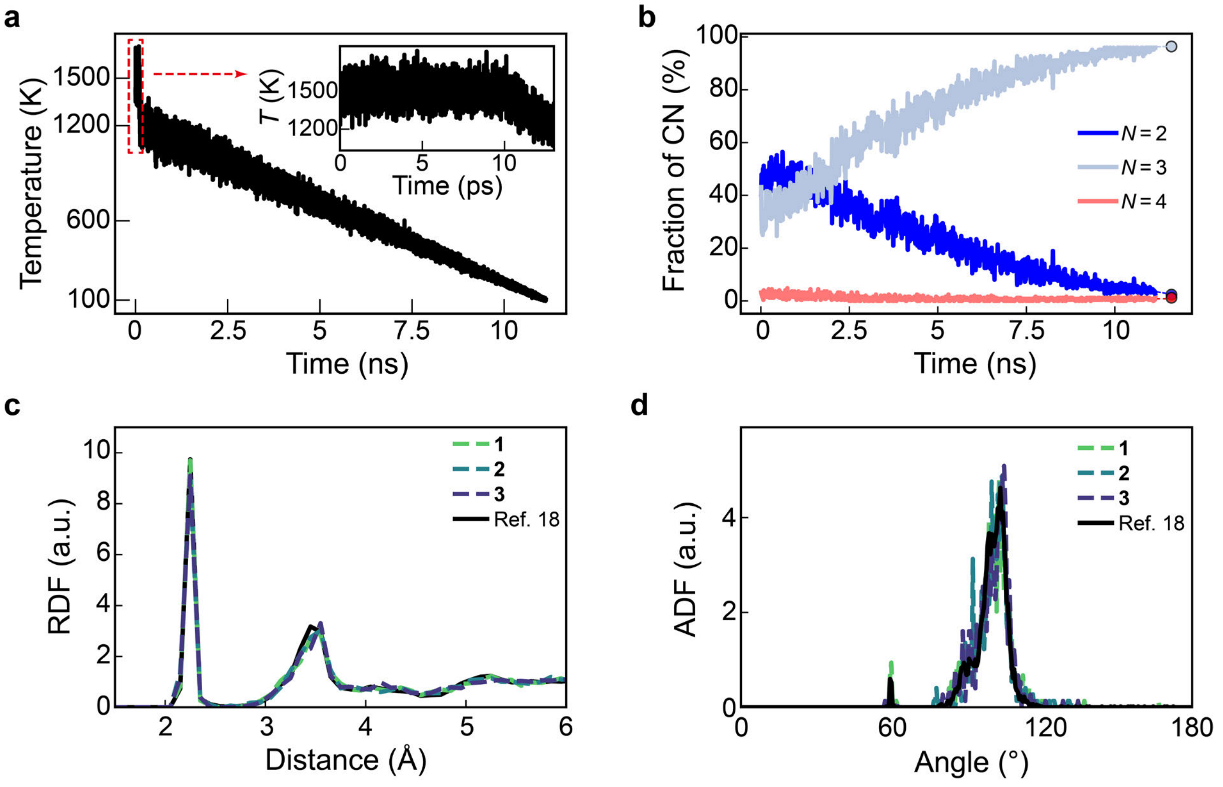

Figure S1. Generating a-P structural models with machine-learning-driven simulations. (**a**) The temperature profile of the melt-quench process used to generate the amorphous models in this work (as described in the Methods section): a simulated disordered liquid phase is cooled over about 10 ns, or 10 million simulation steps. (**b**) Counts of coordination numbers during the melt-quench process. In the liquid phase, most atoms are two- (≈ 49 ± 4%) and three-fold (≈ 29 ± 6%) connected. During quenching, a network of mostly three-fold-connected atoms emerges, and in the final structures (indicated by markers), almost all of the atoms have N = 3 neighbours. (**c**–**d**) Calculated radial distribution function (RDF, panel **c**) and angular distribution function (ADF, panel **d**) for the three pristine models of a-P (**1** to **3**) generated in the present work, compared to those calculated from the larger-scale model in previous work in Ref. [S9] (Ref. 18 in the main text).

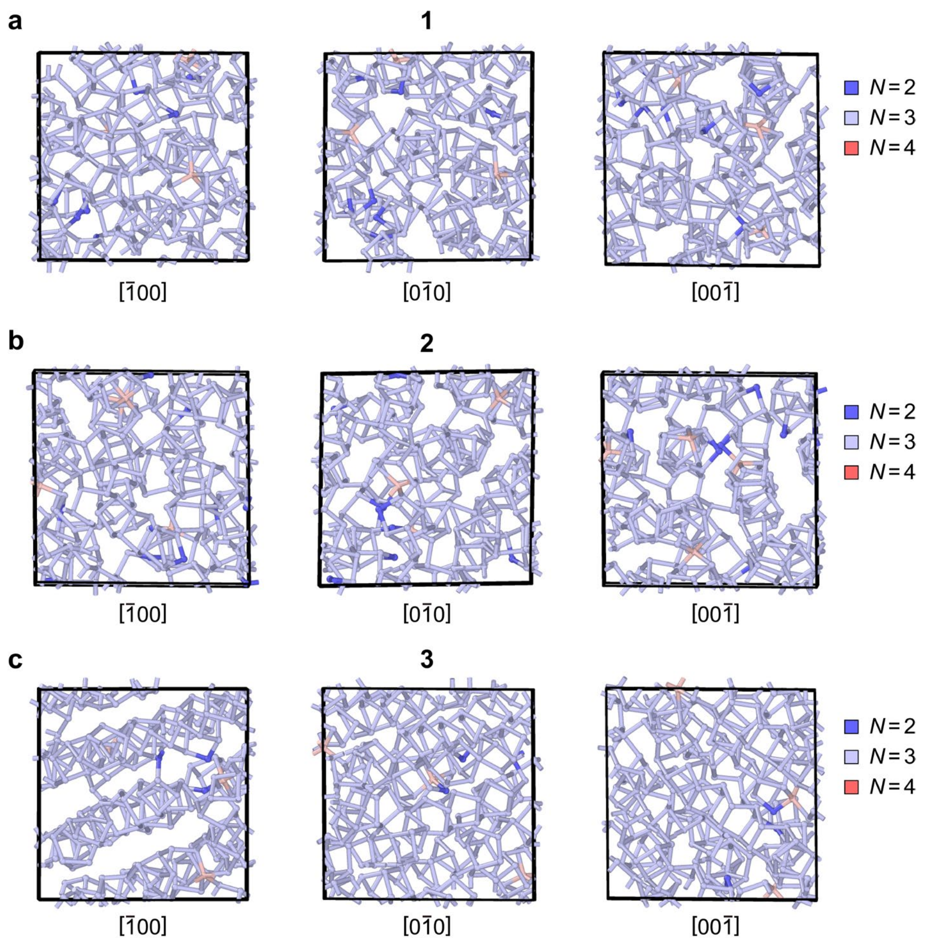

Figure S2. (**a**–**c**) Structures of the three pristine models generated in this work. Views along the $[\bar{1}00]$, $[0\bar{1}0]$, and $[00\bar{1}]$ directions are shown, respectively. Atoms are colour-coded based on the coordination numbers, *viz.* $N = 2$ (blue), $N = 3$ (pale blue), and $N = 4$ (pink), determined by counting atomic neighbours up to a 2.4 Å cut-off.

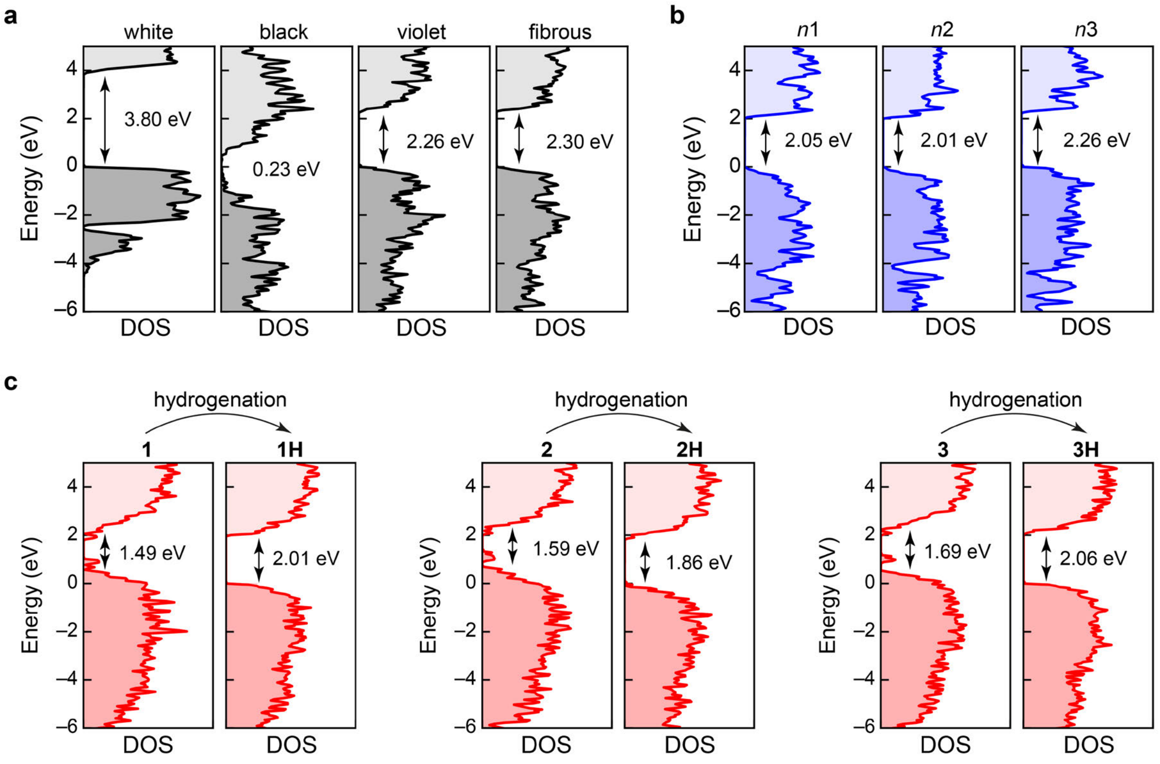

Figure S3. Computed electronic densities of states (DOS, at the HSE06 level) for various phosphorus modifications, including (**a**) crystalline phosphorus allotropes (white, black, violet, and fibrous phosphorus), (**b**) three nanorod models (*n*1 to *n*3) following Bachhuber *et al.*, as described in the main text, as well as (**c**) the pristine and hydrogenated a-P models generated in the present work. The band gaps were computed using the difference between the top of the valence band and the bottom of the conduction band: that is, we omitted the mid-gap defect states when determining band gaps for pristine a-P models. An opening of band gaps in the a-P models upon hydrogenation is evident.

Supplementary References

[S1] V. L. Deringer, M. A. Caro, G. Csányi, *Nat. Commun.* **2020**, *11*, 5461.

[S2] a) A. P. Bartók, M. C. Payne, R. Kondor, G. Csányi, *Phys. Rev. Lett.* **2010**, *104*, 136403; b) V. L. Deringer, A. P. Bartók, N. Bernstein, D. M. Wilkins, M. Ceriotti, G. Csányi, *Chem. Rev.* **2021**, *121*, 10073–10141.

[S3] A. P. Bartók, R. Kondor, G. Csányi, *Phys. Rev. B* **2013**, *87*, 184115.

[S4] V. L. Deringer, D. M. Proserpio, G. Csányi, C. J. Pickard, *Faraday Discuss.* **2018**, *211*, 45–59.

[S5] A. P. Thompson, H. M. Aktulga, R. Berger, D. S. Bolintineanu, W. M. Brown, P. S. Crozier, P. J. in 't Veld, A. Kohlmeyer, S. G. Moore, T. D. Nguyen, R. Shan, M. J. Stevens, J. Tranchida, C. Trott, S. J. Plimpton, *Comput. Phys. Commun.* **2022**, *271*, 108171.

[S6] a) W. G. Hoover, *Phys. Rev. A* **1985**, *31*, 1695–1697; b) S. Nosé, *Mol. Phys.* **1984**, *52*, 255–268.

[S7] W. Shinoda, M. Shiga, M. Mikami, *Phys. Rev. B* **2004**, *69*, 134103.

[S8] a) Y. Katayama, T. Mizutani, W. Utsumi, O. Shimomura, M. Yamakata, K. Funakoshi, *Nature* **2000**, *403*, 170–173; b) G. Monaco, S. Falconi, W. A. Crichton, M. Mezouar, *Phys. Rev. Lett.* **2003**, *90*, 255701.

[S9] Y. Zhou, W. Kirkpatrick, V. L. Deringer, *Adv. Mater.* **2022**, *34*, 2107515.

[S10] L. Pratt, R. E. Richards, *Trans. Faraday Soc.* **1954**, *50*, 670–674.

[S11] a) G. Kresse, J. Hafner, *Phys. Rev. B* **1993**, *47*, 558–561; b) G. Kresse, F. J., *Phys. Rev. B* **1996**, *54*, 11169.

[S12] a) P. E. Blöchl, *Phys. Rev. B* **1994**, *50*, 17953–17979; b) G. Kresse, D. Joubert, *Phys. Rev. B* **1999**, *59*, 1758.

[S13] a) J. P. Perdew, K. Burke, M. Ernzerhof, *Phys. Rev. Lett.* **1996**, *77*, 3865–3868; b) S. Grimme, J. Antony, S. Ehrlich, H. Krieg, *J. Chem. Phys.* **2010**, *132*, 154104; c) S. Grimme, S. Ehrlich, L. Goerigk, *J. Comput. Chem.* **2011**, *32*, 1456–1465.

[S14] a) J. Heyd, G. E. Scuseria, M. Ernzerhof, *J. Chem. Phys.* **2003**, *118*, 8207−8215; b) J. Heyd, G. E. Scuseria, M. Ernzerhof, *J. Chem. Phys.* **2006**, *124*, 219906; c) J. Paier, M. Marsman, K. Hummer, G. Kresse, I. C. Gerber, J. G. Ángyán, *J. Chem. Phys.* **2006**, *124*, 154709.

[S15] a) A. Tkatchenko, R. A. DiStasio, Jr., R. Car, M. Scheffler, *Phys. Rev. Lett.* **2012**, *108*, 236402; b) A. Ambrosetti, A. M. Reilly, R. A. DiStasio, Jr., A. Tkatchenko, *J. Chem. Phys.* **2014**, *140*, 18A508.

[S16] a) S. Maintz, V. L. Deringer, A. L. Tchougréeff, R. Dronskowski, *J. Comput. Chem.* **2016**, *37*, 1030–1035; b) R. Nelson, C. Ertural, J. George, V. L. Deringer, G. Hautier, R. Dronskowski, *J. Comput. Chem.* **2020**, *41*, 1931–1940.

[S17] a) R. Dronskowski, P. E. Blöchl, *J. Phys. Chem.* **1993**, *97*, 8617–8624; b) V. L. Deringer, A. L. Tchougréeff, R. Dronskowski, *J. Phys. Chem. A* **2011**, *115*, 5461–5466.

[S18] A. Stukowski, *Model. Simul. Mater. Sci. Eng.* **2010**, *18*, 015012.

[S19] K. Momma, F. Izumi, *J. Appl. Crystallogr.* **2011**, *44*, 1272–1276.
